# Characteristics and Outcomes of Patients with Self-directed Violence Presenting to Trauma Centers in the United States

**DOI:** 10.5811/westjem.42022

**Published:** 2025-07-18

**Authors:** Gregory Jasani, Garrett Cavaliere, Rana Bachir, Sarah Van Remmen, Mazen El Sayed

**Affiliations:** * University of Maryland School of Medicine, Baltimore, Department of Emergency Medicine, Baltimore, Maryland; † Penn State College of Medicine, Department of Emergency Medicine, Hershey, Pennsylvania; ‡ American University of Beirut Medical Center, Department of Emergency Medicine, Beirut, Lebanon; § University of Maryland School of Medicine, Department of Psychiatry, Baltimore, Maryland

## Abstract

**Introduction:**

Psychiatric conditions are common presentations to the emergency department, and their prevalence has been steadily increasing. Part of this spectrum of presentations is self-directed violence. Self-directed violence involves suicidal acts and non-suicidal self-injuries that can result in serious morbidity and mortality. This study examines characteristics and outcomes of patients who presented to US trauma centers with self-inflicted injuries and identifies factors associated with survival to hospital discharge in this patient population.

**Methods:**

We extracted data in a retrospective, observational manner from the 2020 National Trauma Data Bank (NTDB) 2020. The NTDB includes data from over 900 trauma centers (900/2,294 total trauma centers in the United States, 39.2%). We performed a descriptive analysis of characteristics, injury patterns and outcomes. All variables were tabulated by outcome (died: yes/no). We then conducted a multivariable logistic regression using a stepwise technique to identify factors associated with the patients’ survival to hospital discharge.

**Results:**

A total of 12,824 patients with self-inflicted injuries were included in this analysis. Their median age was 35 years (interquartile range 25–50), and they were mostly males (74.7%) and White (69.6%). Patients were mostly transported by ground ambulance (78.9%) to Level I (60.6%) and Level II (33.5%) trauma centers. Most patients had a pre-existing condition (70.2%). These included mental/personality disorder (48.2%), alcohol use disorder (11.5%), and substance use disorder (17.7%). The most common mechanism of injury was penetrating trauma (71.6%), followed by blunt trauma (18.0%) and burns (1%). Cutting/piercing was the most common penetrating mechanism (60%) compared with firearm-related trauma (40%). Severe injury (Injury Severity Score ≥ 16) was present in 32.8% of patients. A positive alcohol screen and/or a positive drug screen were reported in 30.2% and 31.2% of patients, respectively. Most patients were admitted to hospital (86%). Overall mortality rate at hospital discharge was 21.7%. We identified Important factors associated with survival to hospital discharge in this patient population.

**Conclusion:**

Patients with self-inflicted injuries treated at US trauma centers have high rates of injury severity and a high mortality rate. This study sheds light on the complex and resource-intensive care needed for this vulnerable patient population.

## INTRODUCTION

Suicide is a significant cause of mortality worldwide. In the United States alone, the US Centers for Disease Control and Prevention (CDC) estimated in 2020 that over 45,000 people successfully completed acts of suicide annually and that 1.2 million attempted suicide.^1^,^2^ This makes suicide the 12^th^ leading cause of death domestically. Emergency department (ED) visits related to suicide attempts have also been increasing over recent years based on data from the National Syndromic Surveillance Program.^3^ An additional analysis performed by Ting et al found that the average annual number for these ED-for-attempted-suicides visits more than doubled from 1993–1996 to 2005–2008.^4^

However, not all individuals who engage in acts meant to harm themselves do so with the intent to end their lives. These acts are defined as non-suicidal self-injury (NSSI)^5^. The difference between suicidal acts and NSSI is the intent of the individual; thus, the characterization of suicidal acts compared with NSSI remains ambiguous due to NSSI possibly leading to death.^6^,^7^ Self-directed violence (SDV) is a term that covers both suicidal acts and NSSI. A previously published analysis by Klonsky in *Psychological Medicine* found that the intent behind SDV functioned to alleviate negative emotions, to communicate with others/get attention, or to escape a situation/responsibility.^8^

Previous ED-based studies have examined the prevalence of SDV and the characteristics of these patients presenting to the ED; however, there is limited information regarding the severity of these presentations and their resource needs.^4^, ^9^, ^10^ In a previous analysis of patients presenting to the ED with SDV, Doshi et al found that patients are usually younger (15–19) with average age 31 years, female sex, and Black race with the primary means of SDV being poisoning followed by penetrating (cutting/stabbing) trauma.^9^ An additional analysis performed by Ceniti et al found that patients presenting with SDV have a history of psychiatric conditions, substance use, and lower socioeconomic status.^10^

Similar to previous studies, Klonsky found that SDV was associated with younger age, being unmarried, and with a history of mental health treatment. Other studies found no association with sex or race/ethnicity.^8^. There was also no association with educational history or household income.^8^ One of the common characteristics seen in these patients is an underlying psychiatric condition. Patients who are contemplating or engaging in SDV often have underlying psychiatric illness and would benefit from comprehensive and sustained psychiatric care.^11^ Unfortunately, there is a shortage of mental health clinicians in the US. Currently, over 57 million Americans suffer from a mental illness.^12^ Despite this need, the shortage is expected to get worse with the estimated shortage in 2024 of ≈30,000 psychiatrists.^13^ As these patients are increasingly unable to access outpatient psychiatric resources, they will turn to emergency services. Since EDs are seeing greater numbers of patients seeking care for psychiatric illness, understanding this vulnerable patient population will be crucial in developing best practices for meeting their needs and optimizing clinical outcomes.^14^

**Population Health Research Capsule**

What do we already know about this issue?

*Patients with self-inflicted injuries are a vulnerable population with a high rate of adverse health outcomes*.

What was the research question?

*This descriptive study examined the characteristics and clinical outcomes of patients with self-inflicted injuries who were treated at US trauma centers*.

What was the major finding of the study?

*This study describes important demographic and outcome information for patients with self inflicted injuries*.

How does this improve population health?

*This study serves as a first step to developing best practices and improving mortality for this vulnerable patient population*.

The COVID-19 pandemic also highlighted additional challenges with access to mental health resources as well as increasing prevalence of anxiety/depressive disorder, sleep disorders, grief reactions, and substance use disorder.^15^–^17^ The reason for this is multifactorial^17^–^20^ with social isolation contributing to the pathophysiology of psychiatric disorders and suicidal behavior.^21^ An increase in SDV and suicide rates was, therefore, expected after the COVID-19 pandemic.^22^

Regardless of intent, individuals who engage in SDV represent a unique and vulnerable patient population. A 2012 study performed by Varley et al examined self-harm as an independent risk factor for intensive care unit (ICU) mortality in trauma and burn patients; however, there is a paucity of literature examining the specific injury patterns, injury severity, and outcomes of patients engaging in SDV.^23^ In a 2016 analysis of the National Trauma Data Bank (NTDB), Mathews et al examined data from 2010–2012 to describe the epidemiology, sex-related differences, and mortality of violent suicide attempts presenting to trauma centers.^24^ Additional studies performed by Foote et al and Martain et al specifically evaluated firearm injuries and hanging injury patterns, respectively.^25^,^26^ In this study we sought to add to this literature by examining characteristics of patients presenting to US trauma centers with all types of self-inflicted injuries and to identify factors associated with survival in this patient population.

## METHODS

### Study Design and Setting

We performed a retrospective observational study using the NTDB 2020 dataset of 1,133,053 records. The NTDB is the largest aggregated traumatic data in the US gathering information from over 900 trauma centers. Patients are included in the NTDB if they sustained one or more traumatic injuries with the diagnosis being one of the following *International Classification of Diseases*, *10^th^ Rev, Clinical Modification* (ICD-10-CM) codes: S00–S99, T07, T14, T20–T28, T30–T32, and T79.A1-T79.A9. Furthermore, patients sustaining any of the following ICD-10-CM codes of superficial wounds are excluded from the dataset: S00, S10, S20, S30, S40, S50, S60, S70, S80, and S90. The NTDB encompasses demographic and clinical information, injury data, pre-existing conditions, diagnoses, hospital procedures and events, and ED and hospital outcomes. The definitions of all variables are available in the NTDB dictionary for the database users. An exemption letter from the Institutional Review Board office at the University of Maryland School of Medicine was obtained for using the NTDB de-identified dataset.

### Selection of Participants

**Figure 1:**
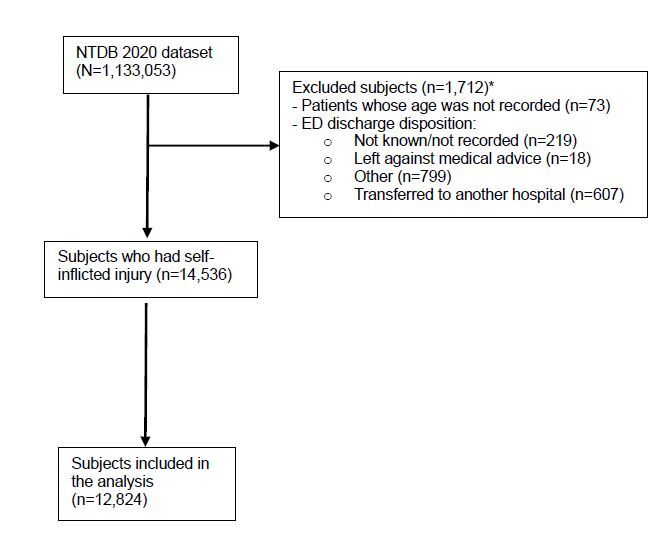
Study participants’ selection from the National Trauma Data Bank 2020. *There are overlaps among the categories of the excluded variables. Some patients with unknown age had as ED disposition one of the excluded categories. These overlaps explain why the final number on which the data analysis was conducted cannot be calculated just by subtracting the number of excluded patients from the selected sample. *ED*, emergency department; *NTDB*, National Trauma Data Bank.

The sample was selected from the variable “injury intentionality” that includes five different responses: 1) unintentional; 2) self-inflicted; 3) assault; 4) undetermined; and 5) other. All patients who had an injury intent as self-inflicted were eligible to be included in the study sample of 14,536. This inclusion minimized the occurrence of any selection bias. Exclusion criteria consisted of 73 patients whose age was not recorded, and 219 with unknown ED discharge disposition (not known/not recorded/not applicable); 18 who left against medical advice: 799 “other” (jail, institutional care, mental health, etc) ; and 607 who transferred to another hospital. A total of 12,824 patients constituted the study sample. We did not calculate the sample size because all eligible patients were pulled from the NTDB database. Figure 1 shows the inclusion and exclusion criteria.

### Data Management and Statistical Analysis

We conducted data management and analyses using the Statistical Package for Social Sciences, SPSS v 27.0 (IBM Corporation, Armonk, NY). For instance, data handling was needed to extract the body region and the nature of injury from all patients’ diagnoses. We carried out descriptive analysis to tabulate the frequencies and percentages of the categorical variables. Age was summarized by reporting its median and interquartile range (IQR) and mean and its standard deviation. Some clinical continuous variables (systolic blood pressure) and ordinal variables (Injury Severity Score, Glasgow Coma Score) were divided into groups based on the adopted categorizations in several peer-reviewed articles. Meaningful recoding for some of the variables (race, mechanism of injury, nature of injury) that have categories with small counts was done with the aim of simplifying the data presentation and interpretation. Variables with missing data >5% (ethnicity, 5.4%; transfusion blood [4 hours], 5.6%; and transfusion platelets [4 hours], 5.6%) were treated by multiple imputation to report accurate estimates. The patients’ demographic and clinical characteristics were stratified by the study outcome (died: yes/no) using the Pearson chi-square or Fisher exact tests for the categorical variables and the Kolmogorov-Smirnov Z test for the age variable.

We conducted a multivariable logistic regression using a stepwise technique to identify the factors associated with the patients’ survival to hospital discharge. All statistically and clinically significant factors were controlled for while carrying out the regression analysis, except for the following surgical procedures that were performed for very few patients: endocrine system, 36 (0.3%;) eye, 242 (1.9%); ear, 174 (1.4%); hemic and lymphatic system, 178 (1.4%); male genital organs, 94 (0.7%); female genital organs, 13 (0.1%); and obstetrical, 2 (0%). In addition, we did not adjust for the trauma type because it conveys some information that can be retrieved from the mechanism of injury through adopting the CDC matrix that presents the trauma type and the injury intentionality of each mechanism of injury. The c-statistic indicated that the final model had an outstanding discrimination between survivors and non-survivors (area under the curve 0.980; *P* value < .001; 95% confidence interval 0.977–0.983]. All tests were interpreted at a predetermined significance level (≤0.05). Of note, we adopted the terms the NTDB uses in its dataset throughout the write-up of the results and the data presentation.

## RESULTS

### Demographics: Age, Sex, and Race

Patients with self-inflicted injuries had a median age of 35 (IQR 25–50) years and were mostly males (74.7%) and White (69.6%). Patients’ basic characteristics are shown in Table 1.

### Method of Arrival and Underlying Mental Illness

Most patients were transported by ground ambulance (78.9%), mainly to Level I (60.6%) and Level II (33.5%) trauma centers. Medicaid/Medicare were the most common payer (41.5%). (Table 1). The majority of patients had a pre-existing condition (70.2%). These include “mental/personality disorder” (48.2%), “alcohol use disorder” (11.5%), and “substance use disorder” (17.7%) (Table 2).

### Mechanism of Injury

The most common mechanism of injury was penetrating trauma (71.6%) followed by blunt trauma (18.0%), with burns (1%) being the least common. Cutting and piercing was the most common mechanism of injury, accounting for 43% of all cases. Firearm injuries were second, accounting for 28%, and falls accounted for 10% (Table 3). In a subgroup analysis of penetrating trauma, cutting/piercing trauma accounted for 60% of cases, while firearm-related trauma accounted for 40% of penetrating cases at a ratio of 3:2.

### Injury Severity

We quantified injury severity using the Injury Severity Score (ISS). Severe injury, defined as an ISS ≥16, was present in 32.8% of all patients with self-inflicted injuries. Nearly 60% of all patients had an open wound on arrival. Approximately 40% had a fracture, and 42% had an internal organ injury. Injuries affected were mainly head/neck (57%), extremities (44.8%), and torso (36.9%) (Table 3).

### Substance Use

Approximately one third of patients had a positive alcohol screen (30.2%), and a positive drug screen was reported in 31.2% of patients. Cannabinoid was most common (17.7%), followed by amphetamines (9.5%), benzodiazepines (7.2%), cocaine (4.4%), opioid (4%), and methamphetamine (3%) (Table 4).

### Disposition

Most patients were admitted to the hospital (86%); 567 (4.4 %) were discharged from the ED, and 1,227 (9.5%) were declared dead in the ED. Of the admitted cohort, 3,513 (27.4%) were sent directly to the operating room, and 3,749 (29.2%) required ICU-level care. For patients admitted to the hospital from the ED, only 3,360 (26.2%) were discharged home; 5,953 (46.4%) required transfer to another facility and 160 (1.2%) left against medical advice. Overall, 1,562 (12.2%) died during their hospitalization. A total of 10,040 patients (78.3%) survived to hospital discharge, and 2,784 patients (21.7%) died in the ED or hospital. Differences between the two groups by outcome (died: yes/no) are presented across the different tables. Results of the multivariate logistic regression analysis are presented in Table 5. We identified important factors positively and negatively associated with survival in patients with self-inflicted injuries.

## DISCUSSION

To our knowledge, this is the first study to examine the characteristics and outcomes of patients with self-inflicted injuries who presented to trauma centers. This study offers insight into key characteristics of this unique and vulnerable patient population. Overall, patients were young with a median age of 35 (IQR 25–50). White was the most commonly reported race, and most were male. This data from the NTDB matches closely with aggregate data regarding suicide from the CDC. Per the CDC, the rates of suicide are highest among middle-aged White men, with men approximately three times more likely to complete suicide compared to women.^2^

This study shows that patients with self-inflicted injuries across trauma centers have very high injury severity and require resource-intensive care. Approximately 33% of patients with self-inflicted injuries had an Injury Severity Score (ISS) of ≥16 on arrival. The ISS is a scoring system used to determine the severity of a traumatic injury, with a score of > 15 considered to be “major trauma.”^27^ In contrast, the study of motor vehicle collision (MVC) victims using the same database found that only 24% of patients had an ISS of ≥16 or greater on arrival.^28^ Of course, there is potentially some overlap of the data between this study and the MVC study as there is evidence that single-occupancy MVCs may be an under-recognized method of suicide.^29^ Similarly, another study examining patients with penetrating trauma reported that only 20% of patients had an ISS of > 15 on arrival.^30^

Patients with self-inflicted wounds also went to the operating room (OR) more frequently than MVC victims: 27% of all patients with self-inflicted wounds had to be taken to the OR compared to only 12% of MVC victims.^28^ Additionally, approximately 30% of all patients with self-inflicted injuries required ICU-level care after presentation to the hospital. In fact, only 4% of patients with self-inflicted injuries were discharged home from the ED. This indicates that the majority of patients who present with self-inflicted injuries will require hospital resources beyond their initial evaluation and stabilization. This has significant implications for ED throughput and resource utilization. Multiple prior studies have shown that patients with psychiatric complaints who require hospitalization have significantly longer lengths of stay in the ED compared to patients with non-psychiatric complaints.^31^–^33^ Additionally, patients with psychiatric complaints often require additional resources such as sitters to maintain safety while in the ED. Their prolonged boarding times also prevents EDs from using those beds to treat other patients seeking care with one study estimating that EDs lose over $2,000 per boarding patient with a psychiatric complaint.^33^

Similarly, even when patients are medically stabilized, 46.4% of these patients are transferred from the hospital where they initiated their medical care. This is likely because these patients, once medically stabilized, also often require psychiatric stabilization best accomplished in the inpatient setting. Unfortunately, with the current shortage of inpatient psychiatric resources, many hospitals are unable to provide that service.^34^ Currently, trauma centers are not required to have inpatient psychiatric capabilities. However, the high rate of patient presentation to trauma centers, both directly and via transfer, raises the question of whether these resources should be more regularly incorporated into trauma centers.

What is perhaps the most striking feature of the data, however, is the high mortality rate among this patient population. Approximately 10% of this patient population die in the ED and trauma bay. Of the patients who survive their initial resuscitation, another 12% will die during their hospitalization. In total, this means that approximately 22%, or more than one in five, patients with a self-inflicted injury will die at some point during their hospitalization. Again, this is in sharp contrast to MVC patients, for whom the mortality rate was ≈2% for both the ED and hospital.^28^ This mortality rate is also higher than all-cause penetrating trauma; only 10% of those patients die in the ED or hospital.^30^

Important factors were found to be associated with decreased survival in this patient population. Increasing age, male sex, and White race were negatively associated with survival. This is supported by data from the CDC and the National Institute of Mental Health showing significant disparities in fatalities across demographic groups, with White males dying by suicide at approximately 2x the rate of Black males, and 3–4x more than Ehite females, and up to 10x more than Black females.^35^,^36^ Increasing clinical severity represented by ISS ≥ 16, systolic blood pressure <90, GCS ≤ 8 or GCS 9–12, and the need for transfusion of blood or platelets within four hours, was negatively associated with survival in this patient population. Similarly, injury resulting from fall or firearm (compared to cut/pierce), internal organ injury, injury to head/neck, and injuries requiring operations to cardiovascular system were also negatively associated with survival. These findings are expected and in line with previous literature examining factors associated with mortality in other trauma populations.^37^ These findings also highlight high-risk injuries that are associated with worse outcomes in patients with self-inflicted injuries and offer evidence for more awareness and prevention campaigns to reduce the heavy burden of this type of trauma.

Rates of mental illness in this country are increasing, and access to outpatient psychiatric care is decreasing. This unfortunate combination means that EDs and trauma centers are likely to see increasing numbers of patients with self-inflicted injuries. This retrospective review suggests that approximately one in five of these patients are not surviving their hospitalization. That number is shocking and should prompt serious discussions across medical specialties about how to lower the mortality rate for this vulnerable patient population. Determining best practices for their care is not only imperative from a resource-utilization perspective but also may be lifesaving.

## LIMITATIONS

The limitations of this analysis are like those of all data registry studies. The quality of the analysis is directly limited by the quality of data reported to the registry. There is variability in the quality of the collected data, an absence of prehospital data, and limited information on complications as well as long-term outcomes. Specific to this analysis, patients were identified by searching for “injury intentionality.” This relies on the coding clinician to add diagnostic codes associated with “self-inflicted.” which may not always be done due to variations in clinician coding habits and the constraints of providing emergency care. Similarly, “self-inflicted” does not allow for determining the patient’s intent as it does not distinguish between a suicide attempt and non-suicidal, self-injurious behavior. It is likely that this analysis under-represents the prevalence of self-directed violence given the dataset’s reliance on diagnostic codes. Additionally, the presence of a concomitant mental health disorder or substance use disorder may be under-represented for the same reason. Thus, the presence or absence of prescribed psychotropic medications as well as medication compliance in the setting of a known mental health disorder is unknown. Neither is the final disposition of these patients (ie, discharge to psychiatric facility vs outpatient psychiatric follow-up vs rehabilitation facilities) known due to the nature of the database. This limits some of the conclusions that can be drawn from this retrospective database analysis.

Additionally, the dataset contains information only for patients who were brought to trauma centers. This inherently does not account for patients with self-inflicted injuries who initially presented to non-trauma hospitals for treatment. Patients transported to trauma centers usually fit the prehospital trauma-triage criteria, which might have biased the selection of a study population with an observed higher mortality. Many patients present with minor injuries to hospitals who do not meet criteria for trauma service activation and, therefore, are not included in the national trauma database. Studies examining self-inflicted injuries and using ED based-registries might report lower mortality rates. The study findings do, however, reflect the complexity of trauma care needed to treat patients with self-inflicted injuries.

## CONCLUSION

Patients with self-inflicted injuries treated at US trauma centers have high rates of injury severity and a high mortality rate. This study sheds light on the complex and resource-intensive care needed for this unique and vulnerable patient population.

## Figures and Tables

**Table 1:** Basic characteristics of patients sustaining self-inflicted injuries.

	Total	Died		P-value
	
	N=12,824	No (n=10,040)	Yes (n=2,784)	
Age (years)				
Mean ± SD	38.65 ± 16.923	37.88 ± 16.027	41.41 ± 19577	<.001*
Sex				
Male	9,581 (74.7%)	7,248 (72.2%)	2,333 (83.8%)	<.001
Female	3,237 (25.2%)	2,787 (27.8%)	450 (16.2%)	
Not known/ not recorded	6 (0%)			
Race				
Black	1,767 (13.8%)	1,454 (14.9%)	313 (11.8%)	
White	8,931 (69.6%)	6,870 (70.3%)	2,061 (77.9%)	<.001
Other Race	1,723 (13.4%)	1,450 (14.8%)	273 (10.3%)	
Not known/ not recorded	403 (3.1%)			
Primary method of payment				
Medicaid/Medicare	5,325 (41.5%)	4,422 (44.9%)	903 (33.4%)	<.001
Self-pay	2,469 (19.3%)	1,568 (15.9%)	901 (33.3%)	
Private/commercial insurance	3,502 (27.3%)	2,806 (28.5%)	696 (25.7%)	
Not billed (for any reason) and other Government and other	1,265 (9.9%)	1,058 (10.7%)	207 (7.6%)	
Not known/ not recorded	263 (2.1%)			
Transport mode				
Ground ambulance	10.124 (78.9%)	7.941 (79.2%)	2,183 (78.4%)	<.001
Helicopter ambulance and fixed-wing ambulance	1,832 (14.3%)	1,258 (12.6%)	574 (20.6%)	
Private/public vehicle/walk-in	629 (4.9%)			
Police and other	220 (1.7%)	617 (6.2%)	12 (0.4%)	
Not known/ not recorded	19 (0.1%)	206 (2.1%)	14 (0.5%)	
Facility level: hospital teaching status				
Community	4,428 (34.5%)	3,442 (35.0%)	986 (36.2%)	<.001
Non-teaching	1,932 (15.1%)	1,458 (14.8%)	474 (17.4%)	
Academic	6,201 (48.4%)	4,939 (50.2%)	1,262 (46.4%)	
Not known/ not recorded	263 (2.1%)			
Trauma Designation Level				
I	7,775 (60.6%)	6,158 (61.4%)	1,617 (58.1%)	.001**
II	4,301 (33.5%)	3,310 (33.0%)	991 (35.6%)	
III	690 (5.4%)	533 (5.3%)	157 (5.6%)	
IV	23 (0.2%)	14 (0.1%)	9 (0.3%)	
Not applicable	15 (0.1%)	8 (0.1%)	7 (0.3%)	
Not known/ not recorded	20 (0.2%)			

* Indicates that the Kolmogorov-Smirnov Z test was used to calculate the P-value.

** Indicates that the Fisher exact test was used to calculate the P-value.

1 Other race is the combination of the following categories: Asian and Pacific Islander, American Indian, and other.

**Table 2:** Presence of underlying illness, psychiatric conditions, and substance use disorders in patients sustaining self-inflicted injuries.

	Total N=12,824	Died		P-value

		No (n=10,040)	Yes (n=2,784)	
Pre-existing Condition				
No	3,431 (26.8%)	2,039 (20.4%)	1,392 (56.8%)	<.001
Yes	8,997 (70.2%)	7,937 (79.6%)	1,060 (43.2%)	
Not known/ not recorded	396 (3.1%)			
Alcohol Use Disorder				
No	10,805 (84.3%)	8,563 (86.4%)	2,242 (94.6%)	<.001
Yes	1,479 (11.5%)	1,352 (13.6%)	127 (5.4%)	
Not known/ not recorded	540 (4.2%)			
Current Smoker				
No	9,022 (70.4%)	6,937 (69.9%)	2,085 (87.9%)	<.001
Yes	3,275 (25.5%)	2,989 (30.1%)	286 (12.1%)	
Not known/ not recorded	527 (4.1%)			
Advanced Directive Limiting Care				
No	12,143 (94.7%)	9,838 (99.3%)	2,305 (97.2%)	<.001
Yes	140 (1.1%)	74 (0.7%)	66 (2.8%)	
Not known/ not recorded	541 (4.2%)			
Attention-Deficit/Hyperactivity Disorder				
No	11,843 (92.4%)	9,515 (96.0%)	2,328 (98.3%)	<.001
Yes	441 (3.4%)	400 (4.0%)	41 (1.7%)	
Not known/ not recorded	540 (4.2%)			
Mental/Personality Disorder				
No	6,125 (47.8%)	4,267 (43.0%)	1,858 (78.3%)	<.001
Yes	6,175 (48.2%)	5,659 (57.0%)	516 (21.7%)	
Not known/ not recorded	524 (4.1%)			
Substance Abuse Disorder				
No	10,015 (78.1%)	7,814 (78.8%)	2,201 (92.9%)	<.001
Yes	2,273 (17.7%)	2,105 (21.2%)	168 (7.1%)	
Not known/ not recorded	536 (4.2%)			

**Table 3:** Severity, mechanism of injury, and nature of injury for patients sustaining self-inflicted injuries.

	Total N=12,824	Died		P-value

		No (n=10,040)	Yes (n=2,784)	
ISS				
≤ 15	8,619 (67.2%)	8,253 (82.2%)	366 (13.1%)	<.001
≥ 16	4,205 (32.8%)	1,787 (17.8%)	2,418 (86.9%)	
GCS Assessment Qualifiers: Patient Chemically Sedated or Paralyzed				
No	11,308 (88.2%)			
Yes	1,516 (11.8%)	9,186 (91.5%)	2,122 (76.2%)	<.001
GCS		854 (8.5%)	662 (23.8%)	
Severe ≤ 8	3,908 (30.5%)			
Moderate 9 – 12	603 (4.7%)	1,365 (14.1%)	2,543 (93.8%)	<.001
Mild 13 – 15	7,881 (61.5%)	554 (5.7%)	49 (1.8%)	
Not known/ not recorded	432 (3.4%)	7,763 (80.2%)	118 (4.4%)	
SBP				
< 90	1,770 (13.8%)			
≥ 90	10,615 (82.8%)	668 (6.8%)	1,102 (44.1%)	<.001
Not known/ not recorded	439 (3.4%)	9,220 (93.2%)	1,395 (55.9%)	
Blood transfusion (4 hours)				
No	10,323 (80.5%)			
Yes	2,501 (19.5%)	8,539 (85.0%)	1,784 (64.1%)	<.001
Transfusion platelets (4 hours)		1,501 (15.0%)	1,000 (35.9%)	
No	12,147 (94.7%)			
Yes	677 (5.3%)	9,680 (96.4%)	2,467 (88.6%)	<.001
Trauma type		360 (3.6%)	317 (11.4%)	
Blunt	2,304 (18.0%)	2,079 (20.7%)	225 (8.1%)	
Penetrating	9,179 (71.6%)	6,913 (68.9%)	2,266 (81.4%)	
Burn	134 (1.0%)	112 (1.1%)	22 (0.8%)	
Other/unspecified	1,207 (9.4%)	936 (9.3%)	271 (9.7%)	
Injury intentionality				
Self-inflicted	12,824 (100%)	10,040 (100%)	2,784 (100%)	
Mechanism of injury				
Cut/pierce	5,538 (43.2%)	5,390 (53.7%)	148 (5.3%)	<.001
Fall	1,398 (10.9%)	1,237 (12.3%)	161 (5.8%)	
Firearm	3,641 (28.4%)	1,523 (15.2%)	2,118 (76.1%)	
Other specified, not elsewhere classifiable	857 (6.7%)	715 (7.1%)	142 (5.1%)	
Other	1,390 (10.8%)	1,175 (11.7%)	215 (7.7%)	
Nature of injury: fracture				
No	7,625 (59.5%)	6,693 (66.7%)	932 (33.5%)	<.001
Yes	5,199 (40.5%)	3,347 (33.3%)	1,852 (66.5%)	
Nature of injury: blood vessel				
No	11,025 (86.0%)	8,447 (84.1%)	2,578 (92.6%)	<.001
Yes	1,799 (14.0%)	1,593 (15.9%)	206 (7.4%)	
Nature of injury: internal organ injury				
No	7,413 (57.8%)	6,723 (67.0%)	690 (24.8%)	<.001
Yes	5,411 (42.2%)	3,317 (33.0%)	2,094 (75.2%)	
Nature of injury: superficial and contusion				
No	9,696 (75.6%)	7,673 (76.4%)	2,023 (72.7%)	<.001
Yes	3,128 (24.4%)	2,367 (23.6%)	761 (27.3%)	
Nature of injury: open wound				
No	5,159 (40.2%)	3,219 (32.1%)	1,940 (69.7%)	<.001
Yes	7,665 (59.8%)	6,821 (67.9%)	844 (30.3%)	
Nature of injury: other specified injury				
No	11,409 (89.0%)	8,791 (87.6%)	2,618 (94.0%)	<.001
Yes	1,415 (11.0%)	1,249 (12.4%)	166 (6.0%)	
Nature of injury: other*				
No	11,147 (86.9%)	8,814 (87.8%)	2,333 (83.8%)	<.001
Yes	1,677 (13.1%)	1,226 (12.2%)	451 (16.2%)	
Body region: torso				
No	8,095 (63.1%)	5,914 (58.9%)	2,181 (78.3%)	<.001
Yes	4,729 (36.9%)	4,126 (41.1%)	603 (21.7%)	
Body region: extremities				
No	7,084 (55.2%)	4,753 (47.3%)	2,331 (83.7%)	<.001
Yes	5,740 (44.8%)	5,287 (52.7%)	453 (16.3%)	
Body region: head and neck				
No	5,508 (43.0%)	5,209 (51.9%)	299 (10.7%)	<.001
Yes	7,316 (57.0%)	4,831 (48.1%)	2485 (89.3%)	
Body region: spine and back				
No	11,544 (90.0%)	8,914 (88.8%)	2630 (94.5%)	<.001
Yes	1,280 (10.0%)	1126 (11.2%)	154 (5.5%)	

*ISS*, Injury Severity Score; *GCS*, Glasgow Coma Score, *SBP*, systolic blood pressure.

**Table 4:** Alcohol and drug screen results on trauma center arrival in patients sustaining self-inflicted injuries.

	Total N=12,824	Died		P-value

		No (n=10,040)	Yes (n=2,784)	
Alcohol Screen				
No	3,514 (27.4%)	2,207 (22.0%)	1,307 (47.0%)	<.001
Yes	9,298 (72.5%)	7,827 (78.0%)	1,471 (53.0%)	
Not known/ not recorded	12 (0.1%)			
Alcohol Screen Result				
Negative screening	6,490 (69.8%)	5,504 (70.3%)	986 (67.0%)	<.001
Mild to moderate	1,814 (19.5%)	1,470 (18.8%)	344 (23.4%)	
Severe	994 (10.7%)	853 (10.9%)	141 (9.6%)	
Drug Screen				
No	8,720 (68.0%)	6,427 (64.6%)	2,293 (82.9%)	<.001
Yes	4,001 (31.2%)	3,529 (35.4%)	472 (17.1%)	
Not known/ not recorded	103 (0.8%)			
Drug Screen: AMP (Amphetamine)				
No	11,603 (90.5%)	8,964 (89.3%)	2,639 (94.8%)	<.001
Yes	1,221 (9.5%)	1,076 (10.7%)	145 (5.2%)	
Drug Screen: Barbiturate				
No	12,738 (99.3%)	9,967 (99.3%)	2,771 (99.5%)	.14
Yes	86 (0.7%)	73 (0.7%)	13 (0.5%)	
Drug Screen: Benzodiazepines				
No	11,896 (92.8%)	9,204 (91.7%)	2,692 (96.7%)	<.001
Yes	928 (7.2%)	836 (8.3%)	92 (3.3%)	
Drug Screen: Cocaine				
No	12,264 (95.6%)	9,550 (95.1%)	2,714 (97.5%)	<.001
Yes	560 (4.4%)	490 (4.9%)	70 (2.5%)	
Drug Screen: Methamphetamine				
No	12,439 (97.0%)	9,698 (96.6%)	2,741 (98.5%)	<.001
Yes	385 (3.0%)	342 (3.4%)	43 (1.5%)	
Drug Screen: Ecstasy				
No	12,756 (99.5%)	9,986 (99.5%)	2,770 (99.5%)	.82
Yes	68 (0.5%)	54 (0.5%)	14 (0.5%)	
Drug Screen: Methadone				
No	12,774 (99.6%)	9,996 (99.6%)	2,778 (99.8%)	.10
Yes	50 (0.4%)	44 (0.4%)	6 (0.2%)	
Drug Screen: Opioid				
No	12,312 (96.0%)	9,561 (95.2%)	2,751 (98.8%)	<.001
Yes	512 (4.0%)	479 (4.8%)	33 (1.2%)	
Drug Screen: Oxycodone				
No	12,713 (99.1%)	9.935 (99.0%)	2,778 (99.8%)	<.001
Yes	111 (0.9%)	105 (1.0%)	6 (0.2%)	
Drug Screen: Phencyclidine				
No	12.776 (99.6%)	9.995 (99.6%)	2,781 (99.9%)	.01
Yes	48 (0.4%)	45 (0.4%)	3 (0.1%)	
Drug Screen: Tricyclic Antidepressant				
No	12.773 (99.6%)	9.991 (99.5%)	2,782 (99.9%)	< .01
Yes	51 (0.4%)	49 (0.5%)	2 (0.1%)	
Drug Screen: Cannabinoid				
No	10.592 (82.6%)	8.099 (80.7%)	2,493 (89.5%)	<.001
Yes	2.232 (17.4%)	1941 (19.3%)	291 (10.5%)	
Drug Screen: Other				
No	12.720 (99.2%)	9.948 (99.1%)	2,772 (99.6%)	.01
Yes	104 (0.8%)	92 (0.9%)	12 (0.4%)	

**Table 5:** Factors positively and negatively associated with survival to hospital discharge in patients sustaining self-inflicted injury, presented as an odds ratio.

	Adjusted odds ratio	95% CI	P-value
Age (years)	0.975 per year	0.969 – 0.981	<.001
Sex [Male]			
Female	1.657	1.289 – 2.131	<.001
Race [Black]			
White	0.561	0.406 – 0.776	<.001
Other Race	0.925	0.615 – 1.390	.71
Primary method of payment [Medicaid/Medicare]			
Self-Pay	0.683	0.516 – 0.905	< .01
Private/Commercial Insurance	1.090	0.850 – 1.398	.50
Not Billed (for any reason) and Other Government and Other	1.013	0.685 – 1.498	.95
Transport Mode [Ground Ambulance]			
Helicopter Ambulance and Fixed-wing Ambulance	1.388	1.069 – 1.803	.01
Private/Public Vehicle/Walk-in	3.228	1.008 – 10.339	.05
Police and Other	1.924	0.568 – 6.518	.29
Pre-existing Condition [No]			
Yes	2.305	1.853 – 2.867	<.001
ISS [≤ 15]			
≥ 16	0.145	0.112 – 0.187	<.001
GCS Assessment Qualifiers: Patient Chemically Sedated or Paralyzed [No]			
Yes	1.992	1.546 – 2.566	<.001
GCS [Mild 13 – 15]			
Severe ≤ 8	0.034	0.026 – 0.046	<.001
Moderate 9 – 12	0.262	0.165 – 0.416	<.001
SBP [≥ 90]			
< 90	0.332	0.257 – 0.429	<.001
Transfusion blood (4 hours) [No]			
Yes	0.674	0.508 – 0.894	.01
Transfusion platelets (4 hours) [No]			
Yes	0.634	0.444 – 0.905	.01
Mechanism of Injury [Cut/pierce]			
Fall	0.482	0.298 – 0.779	< .01
Firearm	0.223	0.160 – 0.312	<.001
Other specified, not elsewhere classifiable	0.850	0.533 – 1.357	.50
Other	0.771	0.510 – 1.166	.22
Alcohol Screen [No]			
Yes	2.067	1.631 – 2.621	<.001
Drug Screen [No]			
Yes	1.746	1.380 – 2.209	<.001
Nature of injury: Internal organ injury [No]			
Yes	0.612	0.471 – 0.795	<.001
Nature of injury: Other [No]			
Yes	0.735	0.541 – 0.997	.05
Body Region: Head and Neck [No]			
Yes	0.417	0.314 – 0.554	<.001
Body Region: Spine and Back [No]			
Yes	1.593	1.106 – 2.294	.01
Body Region: Unclassifiable by body region [No]			
Yes	0.707	0.472 – 1.057	.09
Operations on the nervous system [No]			
Yes	3.197	2.286 – 4.470	.001
Operations on the nose; mouth; and pharynx [No]			
Yes	3.652	2.091 – 6.381	<.001
Operations on the respiratory system [No]			
Yes	1.731	1.347 – 2.225	<.001
Operations on the cardiovascular system [No]			
Yes	0.575	0.458 – 0.722	<.001
Operations on the digestive system [No]			
Yes	3.243	2.427 – 4.334	<.001
Operations on the musculoskeletal system [No]			
Yes	2.592	1.931 – 3.480	<.001
Operations on the integumentary system [No]			
Yes	2.687	2.062 – 3.501	<.001

Brackets in column 1 “[...]” correspond to the reference standard for each comparison.

*CI*, confidence interval; *GCS*, Glasgow Coma Scale; ISS, Injury Severity Score; *SBP*, systolic blood pressure.
